# Interdisciplinary US Hospice Clinician Presence Throughout the Medical Aid in Dying Procedure

**DOI:** 10.1016/j.jpainsymman.2025.11.023

**Published:** 2025-12-05

**Authors:** Todd D. Becker, Karla T. Washington, Elissa Kozlov, Denae J. Gerasta, Grant Yoder Med, Daniel D. Matlock, Stacy M. Fischer, Sara Sanders

**Affiliations:** Division of Public Health Sciences, Department of Surgery, WashU Medicine, St Louis, Missouri; Division of Palliative Medicine, Department of Medicine, WashU Medicine, St Louis, Missouri; Department of Health Behavior, Society, and Policy, Rutgers School of Public Health, Piscataway, New Jersey; Department of Health Systems, Management & Policy, Colorado School of Public Health, Aurora, Colorado; Adult and Child Center for Outcomes Research and Delivery Science, University of Colorado School of Medicine, Aurora, Colorado; Adult and Child Center for Outcomes Research and Delivery Science, University of Colorado School of Medicine, Aurora, Colorado; Department of Health & Behavioral Sciences, University of Colorado Denver, Denver, Colorado; Adult and Child Center for Outcomes Research and Delivery Science, University of Colorado School of Medicine, Aurora, Colorado; Division of Geriatric Medicine, Department of Medicine, University of Colorado School of Medicine, Aurora, Colorado; VA Eastern Colorado Geriatric Research Education and Clinical Center, Aurora, Colorado; Division of Geriatric Medicine, Department of Medicine, University of Colorado School of Medicine, Aurora, Colorado; Division of General Internal Medicine, Department of Medicine, University of Colorado School of Medicine, Aurora, Colorado; University of Iowa School of Social Work, Iowa City, Iowa

**Keywords:** clinicians, end-of-life care, hospice, medical aid in dying, Medicare hospice benefit, presence

## Abstract

**Context.:**

Although hospice policies vary in where they permit staff to be physically located during key stages of the medical aid in dying (MAID) procedure, data needed to inform best practices concerning if and how clinicians themselves are present are lacking.

**Objectives.:**

To (1) assess the sample proportion of clinician presence during each individual stage of the MAID procedure and (2) typologize the distinct trajectories of clinician presence across stages of the MAID procedure.

**Methods.:**

We used secondary cross-sectional survey data from a convenience sample of interdisciplinary US hospice clinicians indicating permissive state and organizational MAID participation policies. Participants reported whether they had ever been present during medication self-administration, after medication self-administration, and after death by indicating if they had been in the same room, in the same residence but not the same room, or never present. We assessed presence and typologized trajectories via frequency and percentage.

**Results.:**

The sample comprised 106 hospice physicians, nurses, social workers, and chaplains. The sample majority reported never having been present during any stage. Across stages, the sample demonstrated 13 distinct trajectories of clinician presence. The most common trajectories illustrated uniformity in physical location across stages. The remaining trajectories reflected transitions in physical locations across stages. Transition subgroups depicted increasing proximity to bedside, increasing distance from bedside, and a combination of both.

**Conclusions.:**

High variability in hospice clinician presence throughout the MAID procedure may differentially affect patient care. Future best-practices research should explore stakeholder experiences of trajectories and stratify trajectories by professional discipline.

## Introduction

In the United States, *medical aid in dying* (MAID) refers to the process that authorizes a terminally ill and otherwise qualified patient to request, receive, and ultimately self-administer a medication prescribed to hasten their death.^1^ As of December 2025, MAID is legally available in 10 states and Washington DC (henceforth, “states”),^1^ which together comprise ≈75 million Americans or 21.9% of the US population.^2^ Despite >100 years of combined implementation experience across legalizing states, understanding of clinician participation across MAID’s clinical pathway—especially during the procedure itself—remains limited.^3–13^ Indeed, the clinical pathway outlined in MAID statutes, which reflects the de facto standard of care,^1^ does not address clinician participation in steps following prescription writing. This lack of knowledge is concerning because statutory protections for conscience-based nonparticipation may affect patient access to legal and goal-concordant care.^14–17^ These potential implications are especially pertinent to hospice care, given that 87.2% of MAID patients receive hospice support prior to death.^18^ In light of anticipated growth in MAID legalization^1,14^ and utilization,^18^ improved understanding of hospice clinician participation during the MAID procedure is needed to develop and evaluate evidence-based best practices.^3^

Specifically, little is known about hospice clinician presence during the MAID procedure.^3,19–23^ Most published data about patient and clinician attitudes are derived from broader studies about hospice clinician MAID participation,^6,10,13,24–29^ suggesting that presence during the MAID procedure is of heightened importance to those clinicians who participate. These studies have found that presence is a hospice patient priority driven by desires for psychosocial^10^ and—in case of noted complications (viz, nausea and vomiting, seizure, prolonged time to death)^13,30^—medical^13,31^ support. The desire to be present is also shared by some hospice clinicians as a means through which to ensure optimal care provision. Previous studies have shown that hospice clinicians have reported overwhelming willingness to be present during the MAID procedure.^3^ Rationales given were grounded most commonly in perceptions that presence would facilitate improved clinical care for both current and future patients.^3^ Hospice clinicians who have been present during the MAID procedure have reported that their provision of support for patients and families^3,10,11,29,31^ ultimately contributed to an enhanced quality of care.^3^

In addition to state policy and personal decisions, clinician presence is informed by hospice organizational policy. Although some hospices permit staff presence throughout the procedure,^23^ many hospices have sought to keep MAID “at arm’s length”^22^ by enacting policies that prohibit staff presence.^7–9,23,32,33^ These prohibitions diverge from statutory permissions for clinician presence,^19,20,22,23^ which have enabled the leading source of evidence-based MAID best practices in the United States to recommend that a clinician be present on the day of the procedure.^34^ Insofar as hospice staff would typically be present on request for non-MAID deaths, these organizational prohibitions highlight a perceived distinction between MAID and non-MAID deaths.^8^ To this end, scholars have contended that prohibitive policies reflect risk management strategies designed to minimize organizational liability stemming from clinician participation—or speculated participation—in activities that would violate the law,^7–9,23,32,33,35,36^ such as clinician administration or coercion.^3^ At the same time, such prohibitions may also challenge hospice care’s purported mission to provide legal, goal-concordant end-of-life care.

Attempts to balance liability with patient care preferences have resulted in a middle-ground approach to hospice clinician presence. These policies have framed the MAID procedure as a process by defining key stages (viz, during medication self-administration, after self-administration, after death) in accordance with physical locations relative to patient bedside permitted for staff presence (viz, in the same room vs in the residence but not the same room; see Sobeck et al,^23^ for examples).^19,20,22,23^ Modulating staff location by stage allows hospices to stipulate greater distances at stages perceived to carry increased liability (ie, during self-administration). By scripting into practice specific patterns of clinician presence throughout the MAID procedure, these “leave-the-room” policies^19,20,22,23,33^ raise implications for care provision that affect patients and clinicians alike. In one extreme case, a hospice agency with a leave-the-room policy fired a nurse for reentering the room at urgent family request to assist a patient who was choking on the medication.^20,22^ Additional evidence has indicated a substantial lack of uniformity in the theoretical trajectories of clinician presence outlined in these policies.^23^ Despite maximizing the provision of permissible hospice support, the presence of high variability in other practice areas has been noted to suggest absent or inconsistent uptake of recommended standards of care.^37^

Despite knowledge of this variability at a policy level,^23^ there is limited knowledge about if and how clinicians are present throughout the procedure in practice. The most robust information source on MAID utilization is annual state report data. Yet the lack of minimum reporting standards across states has been criticized for facilitating disparate and incomprehensive reports of crucial epidemiologic data.^1,38,39^ At present, only California^40^ and Oregon^41^ report on clinician presence during MAID deaths. Whereas both states report whether or not clinicians were present during the stage of self-administration, Oregon^41^ alone reports on presence at time of death. Thus, only 2 of 11 states where MAID is legally available account for clinician presence during the “‘critical’^22^ and defining juncture of MAID delivery,”^21^ and only 1 accounts for clinician presence during the intended clinical outcome. Conversely, no state currently accounts for clinician presence during interim between self-administration and death, nor does any state indicate clinicians’ physical locations during any stage of the procedure. Given that clinician identification is limited to attendees’ roles relative to the MAID case (eg, attending physician, other physician/provider, volunteer),^40,41^ these reports do not indicate whether or not clinicians in attendance are affiliated with a hospice agency. The resulting lack of specificity in the trajectories of hospice clinicians’ physical locations across stages ultimately means that published data do not fully reflect frontline, hospice policy-informed care practices.

Responding to calls to elucidate the depth and breadth of clinician MAID presence,^21^ the purpose of the current study was to map interdisciplinary hospice clinician presence throughout the MAID procedure. Objectives were to (1) assess the sample proportion of clinician presence during each individual stage of the MAID procedure and (2) typologize the distinct trajectories of clinician presence across stages of the MAID procedure.

## Methods

### Study Design

This descriptive study used a cross-sectional design. We drew secondary data from our 1-time, self-administered Qualtrics survey on attitudes toward MAID completed by a convenience sample of interdisciplinary hospice clinicians across the United States.^3,42,43^ Participants had to be ≥18 years old, work as a paid hospice employee, and provide direct patient care to be eligible for inclusion in the broader study. We constructed our sampling frame through partnerships with leading hospice and palliative care professional membership associations (unnamed as per research agreements) representing each core discipline in the Medicare hospice benefit’s definition of the “hospice interdisciplinary group” (ie, medicine, nursing, social work, spiritual care).^44^ We maximized the terms outlined in each association’s research agreement to increase sample recruitment. Doing so prompted variation in the number of contacts (2 vs 5), contact source (association vs research team), and contact mode (email vs newsletter). The data collection period for each association remained open for 30 days from November 2022 through January 2023. We defined surveys with responses to at least half of all items as complete.^45^ We used a raffle mechanism to compensate a random selection of 200 participants with $20 egift cards.

We constructed our surveys with all pertinent, expertrecommended^46^ security features available in Qualtrics.^47^ These efforts notwithstanding, we noted an unusually high influx of responses to our second nursing survey dissemination after only 2 days (n = 2392), leading to our suspicions of bot infiltration. We, therefore, deleted all nursing responses received after this dissemination and conducted a compensatory round of data collection, which generated no concerns of fraud. The Washington University Institutional Review Board approved this study as nonhuman subjects research.

### Measures

We developed our 68-item self-report survey^43^ over 3 iterations. The drafted instrument was reviewed for content and face validity by an expert panel of gerontological researchers with practice experience in aging and end-of-life care (N = 5).^48^ The first author (T.D.B.) then conducted cognitive interviewing of the revised instrument with a typical-case purposive sample of interdisciplinary hospice clinicians (N = 8) to assess item comprehension.^48^ The refined instrument was subsequently pilot tested with another typical-case purposive sample of interdisciplinary hospice clinicians (N = 11) to evaluate technological functionality and survey completion.^48^ These procedures resulted in only minor changes to survey items intended to improve clarity. For the primary measure, specifically, these changes involved more clearly distinguishing among stages of the MAID procedure.

### Hospice Clinician Presence Throughout the Medical Aid in Dying Procedure

Hospice clinician presence throughout the MAID procedure was assessed via a matrix question, asking, “Have you ever been present while a hospice patient used medical aid in dying during any of the following instances?” (see Supplemental Fig. 1). Participants responded to matrix rows identifying key stages of the MAID procedure (while the patient self-administered the means to hasten death, after self-administration has already occurred, after death has already occurred) by selecting among matrix columns indicating physical locations commonly stipulated by hospice policy (yes, I have been present in the same room; yes, I have been present in the same residence but not in the same room; no, I have never been present). This matrix format produced a categorical variable for each individual stage of the MAID procedure. We used these 3 stage-specific variables to construct a composite variable that identified all distinct patterns of physical locations throughout the MAID procedure (theoretical range, 1–27).

### Statistical Analysis

Preliminary analyses included performing descriptive statistics to characterize the sample. To assess sample proportions, we calculated the frequencies and percentages of participant presence during each individual stage of the MAID procedure. To typologize trajectories, we computed frequencies and percentages for each distinct pattern of physical locations throughout the MAID procedure derived through the composite variable. We conducted all statistical analyses in R (Version 4.5.1).

## Results

### Analytic Sample Specification

Our broader survey received a total of 1346 responses. We specified the analytic sample for the current secondary analysis via a series of 3 exclusions. First, we excluded cases that violated a priori study design criteria (850 of 1346 [63.2%]: lack of informed consent provision, 130; study ineligibility, 683; survey breakoff,^45^ 26; Qualtrics security metrics,^47^ 11). Second, we excluded cases based on participant-reported implausibility for MAID presence (388 of 496 [78.2%]; contemporaneous MAID illegality in states within employing hospice’s service area, 362; hospice policy disallowing at least partial employee MAID participation, 26). Third, we excluded cases out of concern for deductive identification (2 of 108 [1.9%]: sparseness in demographic variable categories, 2). These exclusions revealed no missing data in our primary measure, thus yielding our final analytic sample.

### Sample Characteristics

The sample included 106 interdisciplinary hospice clinicians working for hospices that together serviced all states where MAID was legally available at the time of data collection (see Table 1). Age ranged from 26 through 74 years (mean [SD], 53.3 [12.4] years). Participants identified mostly as being men (70 [66.0%]), White (96 of 105 [91.4%]), and Protestant (26 of 103 [25.2%]). Just under half of the sample were physicians (46 [43.4%]), with the other half divided among chaplains (25 [23.6%]), social workers (22 [20.8%]), and registered nurses/advance practice registered nurses (13 [12.3%]). Participants worked in hospice between 1 and 40 years (mean [SD], 11.0 [8.2] years). Three-quarters of the sample reported a generally supportive attitude toward MAID (79 [74.5%]) and nearly the full sample reported ever having received a patient request for MAID (104 [98.1%]). Whereas the vast majority of participants reported ever having provided information to a hospice patient about MAID (100 [94.3%]), slightly fewer participants reported ever having provided end-of-life care related to a hospice patient’s use of MAID (68 [64.2%]). The sample majority further indicated hypothetical willingness to be present throughout the MAID procedure (82 [78.3%]). Most of the hospices for which participants worked were not religiously affiliated (81 of 105 [76.4%]), were not for profit (81 [76.4%]), and had policies permitting only partial MAID participation (70 [66.0%]).

### Sample Proportion of Clinician Presence During Each Individual Stage of the Medical Aid in Dying Procedure

**Fig. 1.**
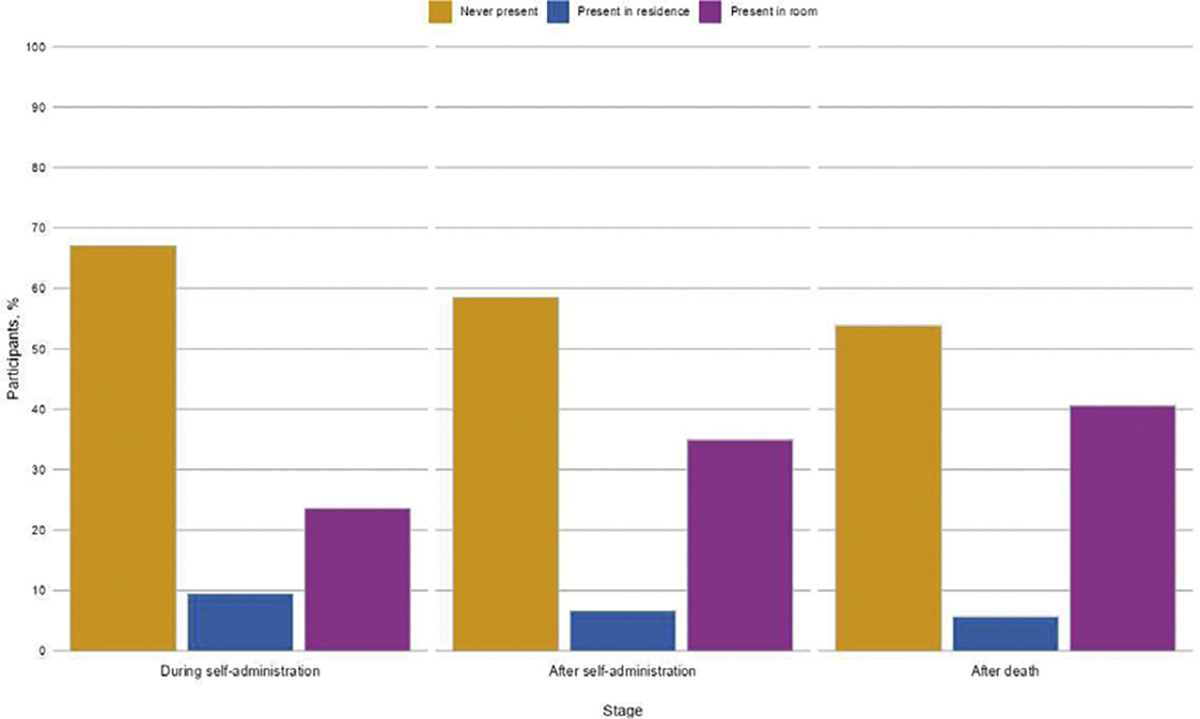
Sample proportions of presence during each individual stage of the medical aid in dying procedure (N = 106).

Descriptive results demonstrated that, for each stage, the most common response was never having been present (during self-administration: 71 [67.0%], after self-administration: 62 [58.5%], after death: 57 [53.8%]; see Fig. 1). The second most common response for each stage was having been present in the same room (during self-administration: 25 [23.6%]; after self-administration: 37 [34.9%]; after death: 43 [40.6%]). Least common for each stage was having been present in the residence but not the same room (during self-administration: 10 [9.4%]; after self-administration: 7 [6.6%]; after death: 6 [5.7%]).

### Distinct Trajectories of Clinician Presence Across Stages of the Medical Aid in Dying Procedure

**Fig. 2.**
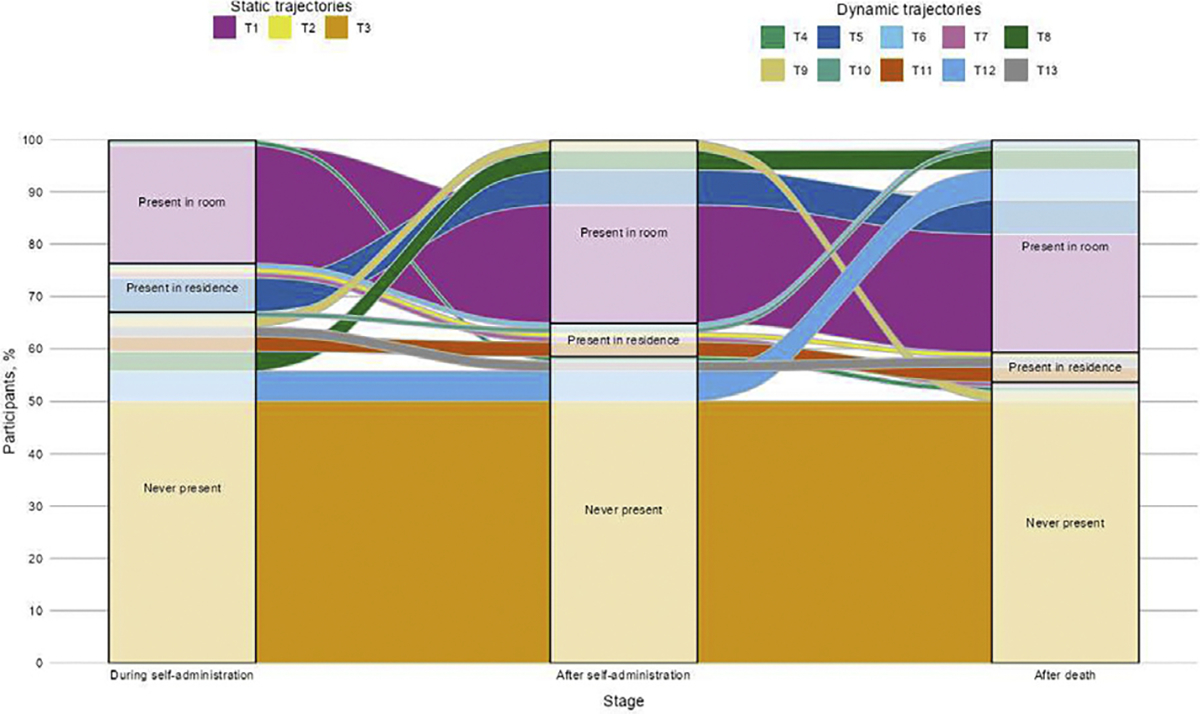
Trajectories of hospice clinician presence across stages of the medical aid in dying procedure (N = 106).

Further descriptive results revealed a total of 13 distinct trajectories throughout the MAID procedure (see Fig. 2). In general, the most common trajectories were characterized by consistent presence in the same location. The single most common trajectory, never having been present at any stage, independently accounted for half of the sample (53 [50.0%]). Reflecting the inverse, the second most common trajectory independently represented roughly one-fifth of the sample and was typified by consistent presence in the room (24 [22.6%]). The midpoint of consistent presence in the residence but not the room was reported by only a single participant (1 [0.9%]).

The 10 remaining trajectories reflected transitions in physical locations across stages. Six of these trajectories demonstrated increasing proximity to the patient. Conversely, 3 trajectories demonstrated decreasing proximity to the patient throughout the MAID procedure and 1 trajectory demonstrated increasing proximity followed by decreasing proximity throughout the MAID procedure.

## Discussion

To our knowledge, these results represent the first attempt at mapping hospice clinician presence throughout the MAID procedure. In so doing, they reflect the first known data-driven acknowledgement of hospice policy in defining the MAID procedure as a multistage process. Leveraging an interdisciplinary sample of clinicians working for hospices servicing all states where MAID is currently legally available, results indicated substantial heterogeneity in presence within and across stages.

Objective 1 results indicated that the sample majority reported never having been present during any stage (range, 53.8%–67.0%). Inasmuch as MAID utilization—despite growing—remains rare,^18^ hospice clinician presence during the procedure itself appears even rarer. Our sample proportions of presence, when summed across physical locations, appear consistent with the limited, available estimates of presence during self-administration (33.0% vs 35.6%–43.3%, respectively)^40,41^ and following self-administration (41.5% vs 36.6%, respectively).^41^

Absent available comparisons, our finding that roughly half of our sample (46.3%) reported ever having been present after death had already occurred represents a novel contribution. Hospice clinicians are typically called to pronounce patient death. Thus, irrespective of noted organizational and/or individual distinctions between MAID and non-MAID deaths,^8^ customary hospice care may prompt some level of clinician involvement—if even indirect—in MAID cases. The novelty of our results is further evidenced by distinguishing our derived sample proportions by key physical locations stipulated by hospice policy. This delineation is noteworthy in that where hospice clinicians are located is likely to affect the support that they can offer. Taken together, these findings suggest opportunities for increased granularity in future data collection efforts to accurately capture frontline care practices. Nevertheless, because our convenience sampling procedure does not support the establishment of population prevalence, it should be noted that our derived sample proportions may not represent true values. This recognition notwithstanding, our findings lay the initial groundwork for needed future efforts.

Objective 2 results revealed a total of 13 distinct trajectories, accounting for just under half of the 27 trajectories theoretically possible through our matrix question (48.1%). These trajectories reflected 2 general patterns based on movement across stages. *Static trajectories* were typified by consistency in presence across stages of the MAID procedure, whereas *dynamic trajectories* were marked by transitions in physical location across stages. These contrasting patterns offer important implications for care provision. Static trajectories, such as uniformity in never being present, may leave patients and families without desired clinical support during critical moments. Conversely, the same flexibility that dynamic trajectories—whether driven by personal, organizational, or other reasons—may offer may be difficult to communicate clearly and consistently, thereby leading to confusion, unmet expectations, and potentially inequitable care. The lack of a standardized approach may also complicate the training of new clinicians, who may subsequently need to navigate considerable complexity in practice without clear or consistent guidelines, potentially affecting their ability to provide optimal care.

Although few in number (3 of 13), static trajectories accounted for three-quarters of the sample (73.6%). Aside from the residence-based trajectory reported by a single participant, static trajectories were concentrated at both extremes of physical locations. That is, participants were either (*a*) not present at all or (*b*) as close to the patient as physically possible. This dichotomy of wholesale adoption or the lack thereof makes sense in light of recent speculations that organizations or individuals already participating in one aspect of MAID-related care may perceive limited additional risk in participating in others.^21^ Accordingly, uniformity in clinician presence throughout the MAID procedure may appear to organizations and staff as more practically implementable.

In contrast, the varied transitions constituting dynamic trajectories resulted in trajectories that were much greater in number (10 of 13) and, therefore, much more sparsely represented in the sample (26.4%). Dynamic trajectories contained 3 subgroups characterized by fluctuations in proximity and/or distance to patient bedside across stages. The most common subgroup depicted a general trend of increasing proximity to patient bedside across stages. Although the basis for these transitions remains unclear, this result aligns with previous perceptions that organizational and clinician risk appear highest while patients self-administer the MAID medication and declines across subsequent stages. Thus, patients and families may receive limited care at stages during which desires for hospice support^10,13,31^ may be pronounced. This finding extends scholarship suggesting that stages punctuating this procedure are not valuated equally from organizational^19,20,22,23^ or clinician^10^ standpoints by showing how and where these differential valuations manifest across the broader process of self-administration.

Across all 13 trajectories, the most common trajectory reflected uniformity in never having been present. This static trajectory was endorsed by half of our sample (50.0%). Inasmuch as clinician presence has been recommended as a best practice,^34^ this finding signals a departure from practices advocated to facilitate optimal care provision. It is also noteworthy for its implications on other identified trajectories: This finding means that the 12 remaining trajectories—all of which involve some type of presence—corresponded to only 53 participants. Observation of such heterogeneity, especially given our modest sample size, indicates a substantial lack of standardization during a formative step in MAID’s clinical pathway.

### Limitations

Several limitations warrant mentioning. First, we recruited our convenience sample through hospice and palliative care professional membership associations. The resulting sampling bias limits the generalizability of our results. Second, some of our partnering associations were unable to subset their membership lists to retain only those members employed in hospice settings (vs nonhospice palliative care). As a result, we were unable to faithfully estimate response rates or evaluate response bias. Relatedly, our maximization of each research agreement’s terms introduced mode effects, which likely prompted variation in response across associations. Last, our results may be limited in 2 ways by the secondary data available. In particular, the lack of measures clarifying the specific activities (eg, presence) prohibited by hospices permitting only partial MAID participation may threaten internal validity. Nevertheless, our finding that half of our sample reported presence in some capacity may attenuate this threat. Additionally, because our measure of presence considered all previous professional experience, we cannot confirm that the trajectory generated for each participant reflects their presence in the same patient case. All the same, this threat to internal validity may be offset by recognition that organizations and clinicians generally appear relatively consistent in their approaches to MAID participation.

### Implications

These results offer ready implications for future policy and research. In terms of policy, we echo previous calls to improve the depth and breadth of data presented in state MAID utilization reports,^1,38,39^ especially regarding hospice clinician presence.^3,21,39^ At present, only 2 states report on clinician presence at all—only 1 of which details presence during more than 1 stage—and none clarifies hospice affiliation of attendees. Expanded reporting, therefore, represents a necessary first step toward cohering state-level reporting practices and organizational policy driving frontline care delivery. To that end, we also echo pushes for increased organizational transparency about MAID participation^23,49^ to enhance informed decision-making about hospice enrollment and facilitate goal-concordant care.^24^ Hospices may support transparency via the public publishing of organizational policies developed in consideration of emerging best practices.^34^

In terms of research, future studies are needed to more fully elucidate the context of hospice clinician presence during the MAID procedure in service of evaluating outcomes and developing best practices. Emerging evidence suggests that clinician engagement across MAID’s clinical pathway may vary across the hospice interdisciplinary group due to time points of heightened, discipline-specific patient needs.^21,50,51^ Thus, future quantitative studies should consider stratifying trajectories of clinician presence during the MAID procedure by professional discipline to inform clinical guidelines. Future qualitative studies should explore how patients and clinicians experience these trajectories to assess support needs and optimize care delivery. Priority foci include perceptions of quality of care and perceptions of relational ethics.

## Supplementary Material

1

## Figures and Tables

**Table 1:** Sample Characteristics (N = 106)

Characteristic	No. (%)

Participant	
Age, mean (SD) [range], y	53.3 (12.4) [26.0–74.0]
Gender	
Man	70 (66.0)
Woman	36 (34.0)
Race (valid n = 105)	
White	96 (91.4)
Person of color	9 (8.6)
Religious identity (valid n = 103)	
Agnostic/Atheist	22 (21.4)
Catholic	9 (8.7)
Jewish	11 (10.7)
Protestant	26 (25.2)
Other Christian	14 (13.6)
Other	21 (20.4)
Professional discipline	
Physician	46 (43.4)
Nurse (registered nurse/advance practice registered nurse)	13 (12.3)
Social worker	22 (20.8)
Chaplain	25 (23.6)
Duration of time working in hospice, mean (SD) [range], y	11.0 (8.2) [1.0–40.0]
General MAID attitude	
Support	79 ( 74.5)
Neither support nor oppose	11 (10.4)
Oppose	16 (15.1)
Ever received a patient request for medical aid in dying from a hospice patient	
Yes	104 (98.1)
No	2 (1.9)
Ever provided information about medical aid in dying to a hospice patient	
Yes	100 (94.3)
No	6 (5.7)
Ever provided end-of-life care related to a hospice patient’s use of medical aid in dying	
Yes	68 ( 64.2)
No	38 (35.9)
Hypothetical willingness to be present throughout medical aid in dying procedure	
Yes	83 (78.3)
Unsure	8 (7.6)
No	15 (14.2)
Hospice	
Religious affiliation (valid n = 105)	
Yes	24 (22.9)
No	81 (76.4)
Tax status	
For profit	25 (23.6)
Not for profit	81 (76.4)
MAID participation policy	
Partial	70 (66.0)
Full	36 (34.0)

## Key Message

Only a minority of our interdisciplinary hospice clinician sample reported ever having been present in the MAID procedure during self-administration, after self-administration, or after death. Combined patterns of physical locations across stages revealed a total of 13 distinct trajectories. Results highlight a substantial lack of standardized approach to care.
