# Supplementary material for: Interdisciplinary US Hospice Clinician Presence Throughout the Medical Aid in Dying Procedure
Source: J Pain Symptom Manage. Author manuscript; Available in PMC 2026 Mar 23. (PMC13007664; doi:10.1016/j.jpainsymman.2025.11.023)
Supplement: 1 [file NIHMS2149445-supplement-1.pdf]

ARTICLE IN PRESS

Appendix

Supplemental Fig. 1. Survey measure used to assess hospice clinician presence throughout the medical aid in dying procedure.

Have you ever been present while a hospice patient used medical aid in dying during any of the following instances?			
	Yes, I have been present in the same room	Yes, I have been present in the same residence but not the same room	No, I have never been present
While the patient self-administered the means to hasten death	○	○	○
After self-administration has already occurred	○	○	○
After death has already occurred	○	○	○
